# The Effect of Learning on the Function of Monkey Extrastriate Visual Cortex

**DOI:** 10.1371/journal.pbio.0020044

**Published:** 2004-02-17

**Authors:** Gregor Rainer, Han Lee, Nikos K Logothetis

**Affiliations:** **1**Max Planck Institute for Biological CyberneticsTübingenGermany

## Abstract

One of the most remarkable capabilities of the adult brain is its ability to learn and continuously adapt to an ever-changing environment. While many studies have documented how learning improves the perception and identification of visual stimuli, relatively little is known about how it modifies the underlying neural mechanisms. We trained monkeys to identify natural images that were degraded by interpolation with visual noise. We found that learning led to an improvement in monkeys' ability to identify these indeterminate visual stimuli. We link this behavioral improvement to a learning-dependent increase in the amount of information communicated by V4 neurons. This increase was mediated by a specific enhancement in neural activity. Our results reveal a mechanism by which learning increases the amount of information that V4 neurons are able to extract from the visual environment. This suggests that V4 plays a key role in resolving indeterminate visual inputs by coordinated interaction between bottom-up and top-down processing streams.

## Abstract 2

In monkeys trained to identify natural images embedded in noise, changes are seen in the information about learned stimuli signaled by V4 neurons

## Introduction

It is well established that learning can have a strong impact on neural responses to visual stimuli in high-level association cortices such as inferior temporal (IT) or prefrontal (PF) cortex, where the activity of single neurons reflects learning in pair association, object identification, or categorization tasks (Sakai and Miyashita 1991; Logothetis et al. 1995; Booth and Rolls 1998; Kobatake et al. 1998; Erickson and Desimone 1999; Rainer and Miller 2000; Freedman et al. 2002; Sigala and Logothetis 2002). In these studies, learning is thought to modify neural activity to represent task-relevant attributes, such as trained views of three dimensional objects (Logothetis et al. 1995) or associations between paired visual stimuli (Sakai and Miyashita 1991; Erickson and Desimone 1999). The learned representations often exhibit invariance for stimulus features such as size (Logothetis et al. 1995), rotation (Booth and Rolls 1998), or stimulus degradation (Rainer and Miller 2000). Similar neural activity to within-category stimuli during categorization (Freedman et al. 2002) can also be thought of as a learning-dependent form of invariance. Several lines of evidence suggest that these learning effects involve synaptic plasticity and thus represent long-lasting modifications to visual association cortices.

Recent evidence suggests that neurons in early visual sensory areas can also modify their response properties with learning. In particular, several studies have revealed learning-related changes in primary visual cortex (V1) (Crist et al. 2001; Schoups et al. 2001; Ghose et al. 2002), although the extent and functional significance of these learning effects remains somewhat controversial (Schoups et al. 2001; Ghose et al. 2002). Available evidence suggests that classical V1 response properties such as receptive field size or orientation tuning parameters are affected relatively little by learning, while learning does appear to cause general reduction in activity for trained stimuli as well as a task-dependent increase in the influence of nonclassical surround stimulation on the neuron's response.

Learning thus appears to affect both low and high level areas of the ventral visual stream. The results obtained by studies in these two areas are, however, difficult to compare directly, owing to substantial differences in experimental design. In studies of IT or PF cortex, studies typically employ ‘complex' visual stimuli such as Fourier descriptors (Sakai and Miyashita 1991), computer-rendered animals (Freedman et al. 2002), or colored photographs and artwork (Erickson and Desimone 1999). These stimuli are generally presented at the center of gaze and can be from 1° up to 10° of visual angle in size. Many studies also include a selection process that determines which of the neurons encountered in a given penetration are chosen for further quantitative study. By contrast, available learning studies in early visual areas follow well-established rules for investigation of primary and extrastriate visual areas. These studies employ ‘simple' visual stimuli such as oriented bars (Crist et al. 2001) or gratings (Schoups et al. 2001; Ghose et al. 2002). These stimuli are generally presented at eccentric locations, with stimulation parameters adjusted to the receptive field and orientation selectivity of the single neuron currently under investigation. Thus, both stimulus type and experimental procedure generally differ substantially, depending on whether a study investigates low-level sensory or high-level associative visual cortex.

**Figure 1:**
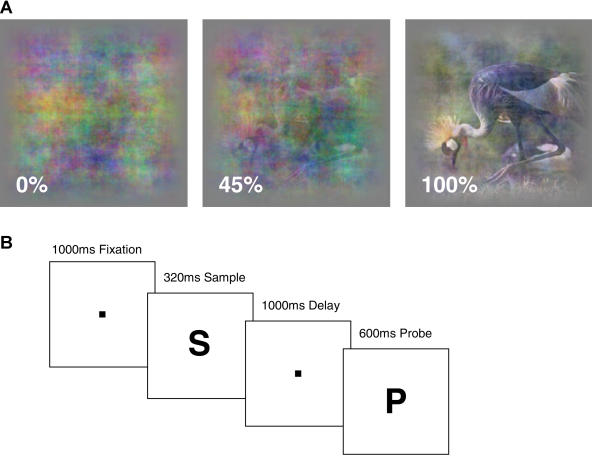
Stimuli and Behavioral Task (A) An example natural image is shown at three coherence levels, corresponding to 100% (undegraded), 45% (degraded), and 0% (pure visual noise). (B) The sequence of trial events for the DMS task used in this study. After a fixation period, a sample stimulus (S) is briefly presented, followed by a delay period and the presentation of a probe stimulus (P). While sample stimuli were presented at different coherence levels, probe stimuli were always presented in undegraded form (100% coherence). The monkeys were required to release a lever if the probe matched the sample.

For a comprehensive account of how learning affects visual processing, the same stimuli and experimental procedure must be used to study different levels of the visual processing hierarchy. What kind of stimuli might be suitable to study visual areas as different as early sensory visual and PF cortex? We decided to use natural images for several reasons: The primate visual system evolved in the natural environment under conditions of ‘natural' stimulation; much is known about their statistical properties and they can therefore be well-controlled; they contain structure at all spatial scales and thus can be expected to activate a large fraction of visually responsive neurons. We avoid subjectively biasing our sample of recorded neurons by always recording from the first neurons whose waveforms we are able to reliably isolate. This ensures that our population of recorded neurons represents an unbiased sample in each brain region under study, and this in turn allows us to compare data obtained from different brain regions. We obtain a sensitive measure of behavioral performance and associated neural activity by employing a stimulus degradation procedure that makes stimuli harder to discriminate by adding various amounts of noise (see Figure 1A). With degradation, stimuli become increasingly indeterminate because all stimuli in a given session are combined with the same noise pattern. Noise is newly generated for every session so that monkeys cannot rely on the specific individual characteristics of a particular noise pattern. Instead, they need to extract task-relevant information from degraded displays, whose particular details vary from day to day. Similarly, outside the laboratory we are rarely presented with familiar stimuli in canonical views and conditions of standard lighting, but instead need to extract this information from complex scenes in which it is embedded. Previously these kind of stimuli were used to study neural activity in the PF cortex (Rainer and Miller 2000), where learning made neural activity more robust to stimulus degradation. After learning, PF neurons tended to fire in a similar manner to undegraded and moderately degraded versions of the same stimulus. Learning thus resulted in a form of neural response invariance, because degradation no longer had an impact on PF neural activity.

Here our aim is to use similar stimuli and behavioral procedures to characterize how learning modifies neural activity in extrastriate visual cortical area V4. Area V4 was chosen because it is considered to be a sensory visual area at an intermediary processing stage in the ventral stream and because it is directly connected to parts of the PF cortex (Petrides and Pandya 1999). Our task was a modified version of delayed-matching-to-sample (DMS) (see Figure 1B). After grasping a metal lever and subsequently attaining central fixation, monkeys viewed a sample stimulus presented at one of six coherence levels ranging from undegraded (100% coherence) to fully degraded (0% coherence). After a brief delay, monkeys were presented with a probe stimulus (always at 100% coherence) and had to release a lever if the probe matched the sample (i.e., if the sample was identical to or was a degraded version of the probe stimulus). During each session, we employed four highly familiar stimuli and four ‘novel' stimuli that monkeys had not seen previously. Great care was taken to ensure that novel and familiar images differed only in terms of their familiarity to the animal (see Materials and Methods). Using novel and familiar stimuli allowed us to ask whether learning had any effect on monkeys' ability to identify degraded and undegraded versions of natural images. Intermixing novel and familiar images in the same session had the additional advantage of allowing us to estimate for each single neuron in our population, whether there were any learning-related changes in the amount of stimulus-specific information these neurons communicated.

## Results

**Figure 2:**
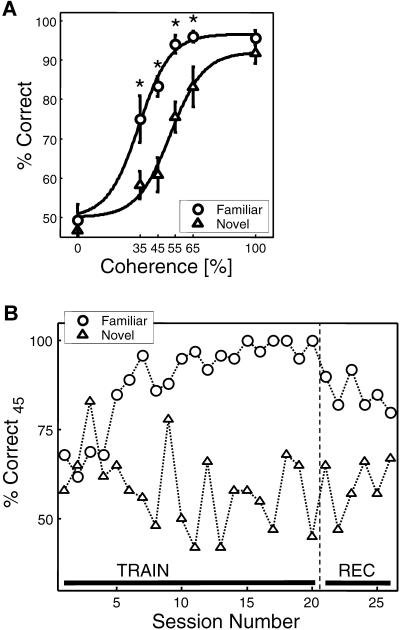
Learning Improved Monkeys' Ability to Identify Degraded Stimuli (A) Behavioral performance for the sessions during which neural data was collected (*n* = 11) is shown as a function of the coherence of the sample stimulus for novel and familiar stimuli. Asterisks denote significant differences in performance for novel and familiar stimuli. (B) The performance at 45% coherence (%Correct_45_) is shown for a set of novel stimuli that is introduced in the first session and then used during all subsequent sessions and thus becomes more and more familiar during subsequent sessions (circles). For comparison, performance with novel stimuli that are new and unique to each session is shown (diamonds). Sessions 1–20 represent purely behavioral training sessions (TRAIN), and sessions 21–26 represent combined behavioral and single unit recording sessions (REC).

We found that learning resulted in significant and robust improvements in monkeys' ability to identify degraded stimuli. Behavioral performance varied systematically with coherence (Figure 2A). Monkeys performed at chance level (50% correct) when stimuli were presented at 0% coherence and thus contained no task-relevant information. For degraded stimuli (35%–65% correct), monkeys performed significantly better with familiar than with novel stimuli (*t*-test, *p* < 0.01). For undegraded stimuli at 100% coherence, the monkeys' performance was near ceiling for both novel and familiar stimuli (92% and 95% respectively; t-test, *p* = 0.12). Learning-dependent performance improvements for degraded stimuli were highly consistent across stimuli and monkeys. There were in fact no significant differences in the monkeys' performance to each of the familiar stimuli across sessions at all coherence levels (one-way ANOVAs, *p* > 0.1), and this was also true for novel stimuli. In addition, performance for novel and familiar stimuli did not differ significantly between the two monkeys at any coherence level (t-tests, *p* > 0.1). Note that the monkeys' excellent perfor-mance with undegraded novel objects reflects the fact that they have acquired the rule of the DMS task and are thus able to perform it near ceiling with novel stimuli. The timecourse of this learning-dependent difference in performance is shown in Figure 2B. Session 1 represents a session in which a set of four initially novel stimuli is arbitrarily chosen and kept constant in subsequent sessions, thus becoming more and more familiar. Comparing performance for these stimuli with performance of novel stimuli that are randomly chosen in each session reveals that it takes several sessions for the learning effect to appear. Performance averaged across the first five session was similar for novel and familiar stimuli (*t*-test, *p* = 0.43). Furthermore, the learning-dependent difference in performance appeared to asymptote after around ten sessions. In summary, learning led to robust improvements in the monkeys' ability to identify degraded natural images while the monkeys performed near ceiling for novel and familiar undegraded images.

**Figure 3:**
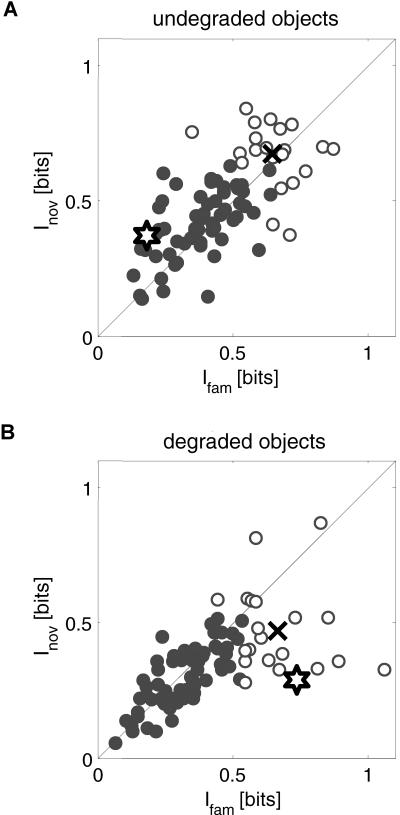
Learning Led to an Increase in V4 Neural Information about Degraded but Not Undegraded Stimuli Here we summarize how much information V4 neurons communicated about novel (I_nov_) and familiar (I_fam_) stimuli for undegraded (A) and degraded (B) stimuli. Each symbol in the scatter plot represents a single neuron and shows how much information this neuron communicated about familiar (x-axis) and novel (y-axis) stimuli. In each scatter plot, white-shaded symbols represent the 25% most informative neurons, i.e., the one-quarter of the population communicating most information about either familiar or novel stimuli. The remaining three-quarters of the population are shown in gray shading. The single neuron example in Figure 5 is represented by the star. The black ‘x' represents the population mean for the 25% most informative neurons.

We now examine whether there were any learning-depen-dent changes in the activity of V4 neurons. Results described in this report are based on a population of 83 V4 neurons. We first asked whether there was any general difference in mean activity elicited by novel and familiar undegraded stimuli. We found that the response of V4 neurons to novel (〈FR_nov_〉 = 36.7 ± 2.8 Hz) and familiar stimuli (〈FR_fam_〉 = 34.2 ± 2.7 Hz) was similar (*t*-test, *p* = 0.14; see also Table 1). Out of the 14 neurons that individually showed a significant difference in activity between novel and familiar stimuli (*t*-test, *p* < 0.05), similar fractions preferred familiar or novel stimuli (6/14 or 43% and 8/14 or 57% respectively; χ^2^ test, *p* = 0.45). We thus found that learning did not lead to a change in the average activity of V4 neurons for undegraded stimuli. Next, we examined whether learning resulted in any change in the amount of stimulus-specific information that V4 neurons communicated. To do this, we computed the mutual information between the set of four familiar or novel stimuli and the associated neural responses (see Materials and Methods). We found that V4 neurons on average communicated similar amounts of information about novel and familiar undegraded stimuli (Figure 3A). The average information communicated by each neuron in the entire population of 83 V4 neurons was similar for novel stimuli 〈I_nov_〉 = 0.48 bits and for familiar stimuli 〈I_fam_〉 = 0.45 bits (*t*-test, *p* = 0.16). We selected 25% of the population (21 out of 83 neurons), which communicated most information about novel or familiar objects (see Materials and Methods). For this population of most informative neurons (white circles in Figure 3A), we also found no difference between novel and familiar stimuli (〈I_nov_〉 = 0.67 bits, 〈I_fam_〉 = 0.65 bits; *t*-test, *p* = 0.48). Thus, for natural images (undegraded stimuli) we saw no significant learning-dependent difference in performance and also no learning-dependent changes in the average activity or in the amount of stimulus-specific information communicated by V4 neurons.

**Table 1:**
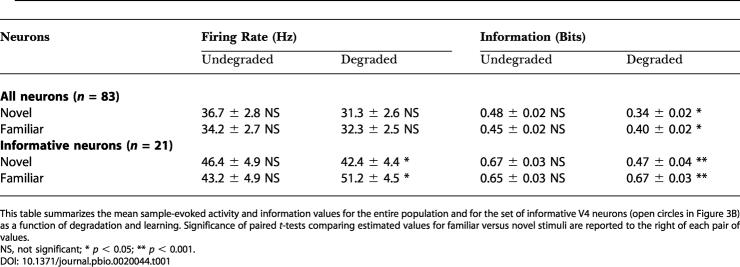
Mean Sample-Evoked Activity and Information Values

This table summarizes the mean sample-evoked activity and information values for the entire population and for the set of informative V4 neurons (open circles in Figure 3B) as a function of degradation and learning. Significance of paired *t*-tests comparing estimated values for familiar versus novel stimuli are reported to the right of each pair of values

NS, not significant; * *p* < 0.05; ** *p* < 0.001

**Figure 4:**
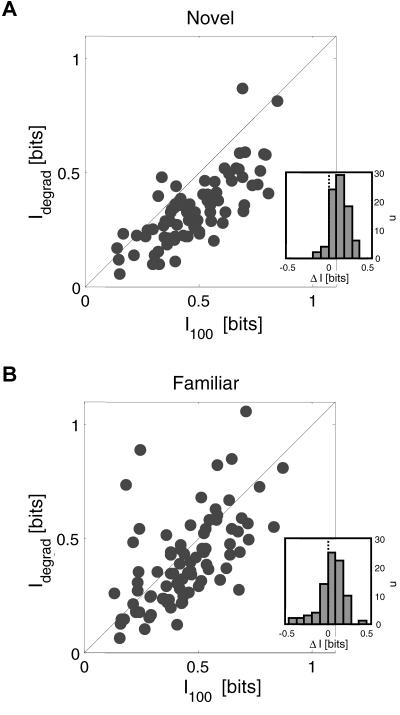
Many Neurons Communicated More Information about Degraded than Undegraded Familiar Stimuli Here we replot the data from Figure 3 to illustrate how much information V4 neurons communicated about degraded (I_degrad_) and undegraded (I_100_) stimuli separately for novel (A) and familiar (B) stimuli. Each symbol in the scatter plot represents a single neuron. The insets show how degradation affected the information communicated by V4 neurons, by plotting histograms of the ΔI distributions (I_100 _− I_degrad_) for novel and familiar stimuli. While 25 neurons (30% of the population) communicated more information about degraded than undegraded familiar stimuli, only six neurons (7% of the population) did so for novel stimuli.

At intermediate coherence levels, the monkeys' ability to correctly identify degraded stimuli was improved by learning, and we asked whether this behavioral improvement was associated with any changes in the activity of V4 neurons. We found that V4 neurons indeed communicated significantly more information about degraded familiar than about degraded novel stimuli (Figure 3B). Considering the entire population, learning led to a significant increase in information about degraded stimuli from 〈I_nov_〉 = 0.34 bits to 〈I_fam_〉 = 0.40 bits (*t*-test, *p* < 0.05). For the 25% most informative neurons (white circles in Figure 3B), we observed an even larger change from 〈I_nov_〉 = 0.47 bits to 〈I_fam_〉 = 0.67 bits (*t*-test, *p* < 0.001), corresponding to a 40% increase in information with learning. We further characterized this effect by examining how degradation affected the amount of information separately for novel and familiar stimuli. For both novel (Figure 4A) and familiar (Figure 4B) stimuli, V4 neurons communicated on average more information about undegraded (I_100_) than degraded (I_degrad_) stimuli (paired *t*-tests, *p* < 0.001), reflecting the fact that behavioral perfor-mance was better for undegraded than degraded stimuli (see Figure 2A). The ΔI distributions (I_100 _− I_degrad_) for familiar and novel stimuli shown in the insets (Figure 4A and 4B), however, differed significantly (paired *t*-test, *p* < 0.001), and learning was associated with a rightward shift in this distribution (〈ΔI_fam_〉 = 0.06, 〈ΔI_nov_〉 = 0.13). Interestingly, the kurtosis or skewness of the ΔI distribution changed by an order of magnitude from 0.13 for novel stimuli to 5.5 for familiar stimuli, similar to experience-dependent effects that have been observed on hippocampal place cell activity (Mehta et al. 2000; Mehta 2001). As a consequence of these learning-dependent changes, many V4 neurons actually communicated more information about degraded than undegraded familiar stimuli (25/83 or 30%), whereas only a small minority did so for novel stimuli (6/83 or 7%). This difference in proportions was significant (χ^2^ test, *p* < 0.001). Taken together, learning accordingly resulted in an increase in the amount of information communicated by V4 neurons about degraded stimuli and many neurons actually communicated more information about degraded than undegraded familiar stimuli.

**Figure 5:**
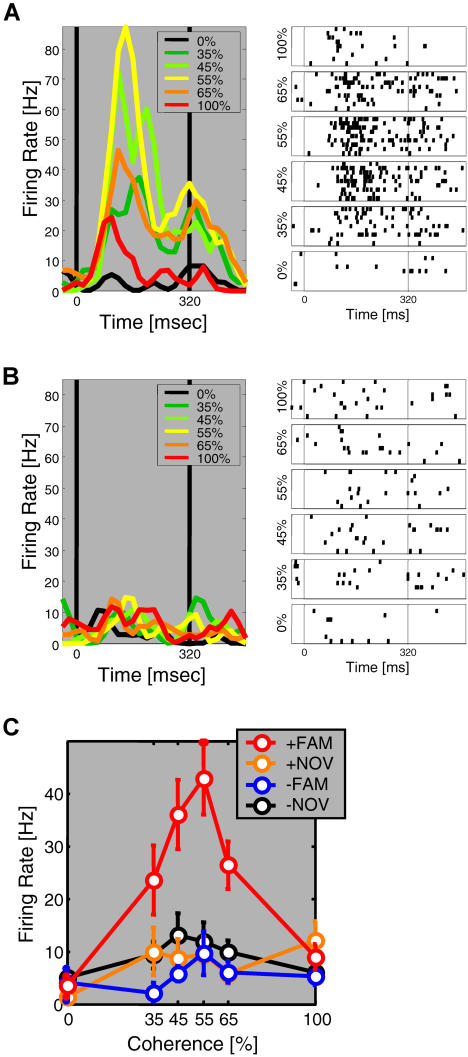
Learning-Dependent Enhancement for Degraded Stimuli—Single Neuron Example (A and B) The activity for an example neuron for its preferred (A) and nonpreferred (B) familiar stimulus is shown in peri-stimulus-time-histogram (PSTH) and raster format. (C) The average firing rate during stimulus presentation as a function of coherence is summarized for this neuron for its preferred (+) and nonpreferred (−) familiar (fam) and novel (nov) stimuli.

**Figure 6:**
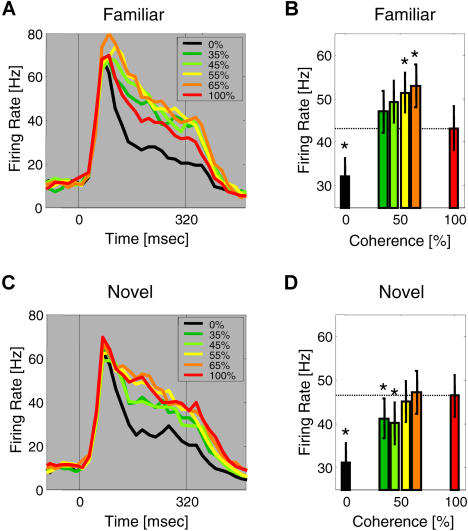
Learning-Dependent Enhancement for Degraded Stimuli—Population Activity These panels show the activity of neurons that communicated most information about degraded stimuli (i.e., white-shaded symbols in Figure 3B) as a function of degradation for familiar (A and B) and novel (C and D) stimuli. The preferred stimulus was used for each neuron. The left column shows activity in PSTH format and the right column shows the mean stimulus-evoked activity at each coherence level; asterisks denote significant differences between activity at each coherence level and activity to undegraded stimuli at 100% coherence (paired *t*-tests, *p* < 0.05).

How did single V4 neurons mediate this learning-depen-dent increase in information about degraded stimuli? The activity of an example neuron is shown in Figure 5 in histogram and raster format for its preferred and nonpreferred familiar stimulus. This neuron showed little or no response to pure visual noise (0% coherence) or to its nonpreferred stimulus at any coherence level (Figure 5B). It was activated to a peak firing rate of about 20Hz by its preferred stimulus (red curve in Figure 5A). Degradation of the preferred stimulus resulted in brisk activity of this neuron, and activity was greater to the preferred stimulus at all intermediate coherence levels (35%–65%) than to the undegraded preferred stimulus (paired *t*-tests, *p* < 0.01). For this neuron (see star in Figure 3), degradation resulted in a large increase in information about familiar stimuli from I_100_ = 0.18 bits to I_degrad_ = 0.74 bits. This example neuron thus displayed a nonmonotonic, inverted U-shaped response as a function of degradation. The responses of this neuron for the preferred and nonpreferred familiar stimuli and also for the corresponding novel stimuli are summarized in Figure 5C. While the preferred novel undegraded stimulus also activated the neuron, degradation of this stimulus was not associated with significant response enhancement. To examine whether the inverted U-shaped response was in fact characteristic of the V4 neurons that communicated most information about degraded stimuli, we plotted the activity of the neurons which were highly selective for degraded stimuli (see white circles in Figure 3B), as a function of coherence, using the preferred stimulus for each neuron (Figure 6). We found that across this population, neural activity was indeed significantly enhanced for familiar stimuli at intermediate coherence levels of 55% and 65% relative to activity to undegraded familiar stimuli (paired *t*-tests: *p* < 0.05). By contrast, activity to novel stimuli systematically decreased with degradation and was significantly below activity to undegraded stimuli at coherence levels of 35% and 45% (paired *t*-tests, *p* < 0.05). As expected, V4 neurons generally showed greater activity to novel and familiar stimuli than to pure noise at 0% coherence (paired *t*-tests, *p* < 0.05). As detailed in Table 1, mean activity was similar for undegraded familiar and novel stimuli, but significantly greater for degraded familiar than degraded novel stimuli (paired *t*-test, *p* < 0.05). Taken together, learning resulted in an increase in information communicated by V4 neurons about degraded or indeterminate stimuli. This increase in information was mediated by neurons that showed an enhancement in neural activity to degraded compared to undegraded familiar stimuli.

**Figure 7:**
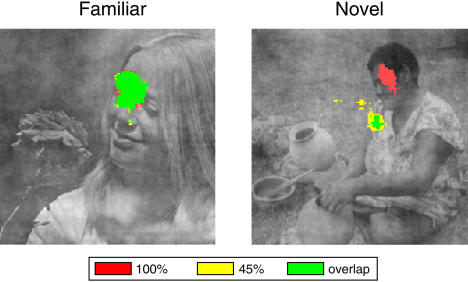
Eye Movement Analysis during Free Viewing Regions of high fixation probability during free viewing of an example familiar and novel stimulus are shown. Monkeys viewed stimuli at 100% coherence (red-shaded regions) and at 45% coherence (yellow-shaded regions). The green-shaded regions represent regions with high fixation probability at both 45% and 100% coherence.

We performed additional behavioral experiments to assess whether learning led to any changes in fixational eye movements, because such changes might shed light on what mediates monkeys' behavioral advantages for familiar degraded stimuli. In these studies, we allowed the monkeys to freely view sample stimuli during task performance and then estimated a fixation probability map (FPM) for each familiar and novel stimulus presented at 45% and 100% coherence (see Materials and Methods. We applied a threshold to this map to identify regions where monkeys tended to fixate with high probability. The thresholded FPMs for 45% and 100% coherence versions of an example familiar and novel stimulus, along with the overlap between these regions, are shown in Figure 7. As can be seen, there was substantially more overlap between the regions of focused eye position at 45% and at 100% after learning. This effect was significant across sessions and stimuli: On average, the overlap region increased by a factor of 2.8 from 0.54 ± 0.14 dva^2^ (degrees of visual angle squared) for novel stimuli to 1.47 ± 0.16 dva^2^ for familiar stimuli (unpaired *t*-test, *p* < 0.0001). There were also significant learning-dependent increases in the high-probability FPM areas at 45% and 100% coherence (at 45% from 1.04 ± 0.25 dva^2^ to 1.88 ± 0.19 dva^2^, unpaired *t*-test, *p* < 0.01; at 100% from 0.84 ± 0.21 dva^2^ to 1.74 ± 0.21 dva^2^, unpaired *t*-test, *p* < 0.01). This learning-dependent increase in the high probability FPM regions and their overlap was highly consistent across sessions and monkeys, and we observed it during all six sessions in both monkeys. Note that the lower FPM values for novel stimuli indicate that eye position was less focused and therefore more distributed before learning, whereas for familiar stimuli robust regions of focused eye position developed.

## Discussion

V4 neurons are generally conceptualized as detectors of visual features of intermediate complexity, such as non-Cartesian gratings (Gallant et al. 1996) or contour features (Pasupathy and Connor 1999). We have found that learning does not affect how V4 neurons respond to undegraded natural images, both in terms of mean firing rate and information communicated about these stimuli. This absence of learning-dependent differences suggests that this V4 selectivity for features of intermediate complexity is not modified by learning, at least during the several weeks of training in the adult monkey during our task. Basic response properties of V4 neurons thus appear not be altered by learning, similar to findings in V1 that have found that parameters such as receptive field size or orientation tuning width remain unchanged even after extensive training (Crist et al. 2001).

Learning does however lead to robust changes in how V4 neurons respond in the presence of degradation. For novel stimuli, V4 neurons tend to act as simple passive feature detectors for which the addition of increasing amounts of noise to the display results in successive reduction in neural activity. Consistent with this finding, we observed a systematic decrease of blood-oxygen level-dependent (BOLD) levels with decreasing stimulus coherence in area V4 of anesthetized monkeys using novel stimuli (Rainer et al. 2001) . After learning, many V4 neurons showed increased activity with the degradation of familiar stimuli, suggesting that they were specifically recruited for difficult discriminations involving the processing of these indeterminate visual inputs. The extraction and amplification of task-relevant elements from visual scenes is a key problem of intermediate-level vision. Our results suggest that V4 neurons play a crucial part in resolving indeterminate visual stimuli and signaling the presence of salient stimulus features. Consistent with this interpretation, studies have found that deactivation or ablation of V4 in monkeys has little impact on basic visual functions, but severely affects shape discrimination (Girard et al. 2002), the identification of images that are occluded or have incomplete contour information (Schiller 1995) or the visual selection in the presence of salient distracters (De Weerd et al. 1999). A recent study found severe deficits after V4 ablation in tasks that required making judgments about oriented line segments embedded in distracter arrays (Merigan 2000), a task that has many similarities to the extraction of task-relevant features from degraded displays in our study. We suggest that lesion-induced deficits are a result of disrupting the operation of V4 neurons which are engaged in selective amplification of task-relevant elements of the visual scene. This idea is consistent with our analysis of eye movements, because monkeys focused more reliably on particular stimulus features for familiar than for novel stimuli. This raises the possibility that allocation of focused attention during task performance under central fixation might have contributed to our results, since attention can greatly enhance the response of V4 neurons to visual stimulation (Moran and Desimone 1985; Connor et al. 1997). Indeed, we suggest that the enhancement in activity and information about degraded familiar stimuli can be conceptualized as a learning-dependent form of attention.

Our findings in V4 are in stark contrast to data obtained in the PF cortex using similar task and stimuli (Rainer and Miller 2000). In the PF cortex, learning resulted in qualitatively different changes in neural activity. Learning resulted in a robust reduction in average neural activity to undegraded stimuli in PF cortex, whereas we found no general differences in activity in V4. This implies that while PF cortex may play a particularly important role in processing novel stimuli (Ranganath and Rainer 2003), extrastriate visual areas communicate feature-specific information largely in the absence of learning-related changes for easy-to-discriminate stimuli. Learning led to neural response invariance across degradation in the PF cortex: neurons that responded differentially to two stimuli maintained this response difference for degraded stimuli after learning, whereas the difference in neural response collapsed with degradation for novel stimuli. Response invariance across degradation implies that the PF cortex does not differentiate between degraded and undegraded versions of a stimulus. Learning thus builds response invariance in the PF cortex. In V4, we found that learning led to a selective enhancement of activity for degraded stimuli over and above the response for undegraded stimuli. While PF neurons showed invariant activity, V4 neurons showed inverted U-shaped noise tuning and were thus most active during difficult discriminations, showing responses consistent with selective amplification of feature-specific activity. Our results suggest that the enhancement observed in V4 may be instrumental in establishing invariance in PF cortex and that interaction between these areas may be required to maintain it. Further experiments using simultaneous recordings from both regions are needed to directly test such a hypothesis. Several studies have identified learning-dependent increases in BOLD signals in extrastriate and temporal visual areas (Dolan et al. 1997; Grill-Spector et al. 2000). Because BOLD measures aggregate activation across many neurons, these studies cannot dissociate whether learning-dependent increases are due to building of invariance or selective enhancement of a subpopulation of neurons. This kind of question is certainly important for characterizing functional properties of brain regions and can be answered definitively only by detailed comparison of neural population activity with simultaneously acquired BOLD signal (Logothetis et al. 1999, 2001).

The task dependence of learning effects in V1 (Gilbert 1998; Gilbert et al. 2001) has been taken as evidence that top-down modulation plays an important role in the learning-dependent modifications seen in V1 neurons and that, accordingly, these changes are reflections of plasticity in higher areas of the visual system. Our findings are certainly consistent with this view and suggest that vision is an active process involving recurrent interaction of different brain regions rather than a purely feed-forward process (Thorpe et al. 1996), although our data are consistent with largely feed-forward processing for familiar undegraded stimuli. A possible biophysical mechanism for this interaction was identified by a recent study, which demonstrated that subthreshold activation of the distal apical dendrite of layer V pyramidal neurons can greatly enhance their response to more proximal inputs (Larkum et al. 1999). Because feedback projections from higher cortical areas tend to arrive in upper cortical layers, this represents a mechanism by which feedback could exert control over activity in sensory cortices (Siegel et al. 2000) and thus contribute to the inverted U-shaped responses observed in the present study.

Several computational models have investigated how brain regions might interact during stimulus identification. A key feature of such models is the interaction between bottom-up and top-down processing (Carpenter and Grossberg 1987; Ullman 1995). Consider a neuron in an intermediate visual area such as V4, receiving bottom-up feature-tuned visual input from visual areas lower in the hierarchy and top-down feedback from higher areas representing possible interpretations of the stimulus. It has been hypothesized that a match between top-down and bottom-up inputs could result in elevated activity or nonlinear response enhancement. We have observed such enhancement for familiar but not for novel stimuli, indicating that learning plays a critical role in facilitating interaction between top-down and bottom-up processing streams. Another type of model has suggested that top-down feedback may represent a predictive code, where top-down signals effectively cancel predictable responses in the bottom-up signal (Mumford 1992; Rao and Ballard 1999). In this scheme, activity would be reduced for undegraded stimuli because it can be accurately ‘predicted away' by higher level areas. Degraded stimuli containing noise might not be accurately predicted, leaving more remaining activity compared to undegraded images. However, based on this model, one would predict lower activity for familiar than for novel degraded stimuli, because more of the familiar stimuli can be predicted away—exactly the opposite of what we have found. Thus, our results are more consistent with theories that conceptualize top-down feedback as high-level stimulus interpretations rather than as an error signals.

## Materials and Methods

### 

#### Behavioral and electrophysiological methods.

Two adult male rhesus monkeys (Macaca mulatta) participated in these experiments. All studies were approved by local authorities and were in full compliance with applicable guidelines (EUVD 86/609/EEC) for the care and use of laboratory animals. Stimuli were 10° × 10° in size, 24-bit color depth, and presented at the center of gaze on a γ-corrected 21-inch monitor with linear display characteristics placed at a distance of 97 cm from the monkeys. Stimuli were generated using Fourier techniques that have been described in detail elsewhere (Rainer et al. 2001). In brief, a large set of natural images was first normalized to have identical Fourier amplitude spectra. Degraded versions of natural images were generated mixing the Fourier phase spectra of natural images with a random phase spectrum corresponding to visual noise, independently for each of the RGB color channels. A different random phase spectrum was used during each session, and it was mixed with all images used during that session.

Each trial began when the monkey grasped a lever and then acquired fixation on a central fixation point. After 1000 ms, a sample stimulus was presented for 320 ms, which could be any one of eight different images at six coherence levels (0%, 35%, 45%, 55%, 65%, and 100%). After a delay of 1000 ms, a probe stimulus was presented for 600 ms, which could be any one of the eight undegraded images (100% coherence). The monkeys were required to release the lever if the probe matched the sample (i.e., if the sample had been identical to or a degraded version of the probe). In case of a nonmatch, a second brief delay (200 ms) followed the probe, and this delay was always terminated by the presentation of the correct matching stimulus, ensuring that monkeys had to make a behavioral response on every trial. The monkeys were rewarded with apple juice for making correct responses and were rewarded randomly at 0% coherence where the sample contained no task-relevant information. During each session, the monkeys performed the task with a set of four familiar stimuli, with which they had many weeks of practice, as well as with a set of four novel stimuli that they had never seen before. Matches occurred on 50% of trials; the other 50% were non-matches selected randomly from the remaining stimuli.

Owing to the normalization procedure, familiar and novel stimuli did not differ in terms of low-level characteristics of spatial frequency content and image intensity. Familiar stimuli from four categories were used (faces, flowers, birds, and landscapes), and one of the four novel stimuli also came from each of these categories. Fixation was monitored with a scleral search coil and sampled at 200Hz (CNC Engineering, Enfield, Connecticut, United States), and the monkeys were required to maintain fixation within a ±1.25° window at all times during the trial. The monkeys completed at least ten trials per condition during each session.

Recordings were made from V4 using standard electrophysiological techniques. We employed a grid system (CRIST, Damascus, Maryland, United States) with eight tungsten microelectrodes (FHC Inc., Bowdoinham, Maine, United States). Preoperative magnetic resonance imaging (MRI) was used to identify the stereotaxic coordinates of V4, which was then covered by a recording chamber. To ensure an unbiased estimate of neural activity, we made no attempt to select neurons based on task selectivity. Instead, we advanced each electrode until the activity of one or more neurons was well isolated and then began collecting data. Comparison of the monkeys' performance during the last six training sessions to performance during recording sessions revealed that performance was unchanged for novel objects (*t*-test, *p* = 0.87), but significantly lower during recording sessions for familiar stimuli (*t*-test, *p* < 0.01), likely owing to nonspecific factors such as additional wait periods during these sessions.

#### Eye movement analysis.

To determine whether there were any learning-related changes in the monkeys' fixational eye movements, we performed separate behavioral experiments in which we allowed the monkeys to freely view the sample stimulus for a period of about 2 s. As before, we presented four familiar and four novel stimuli during each session, but we only used two coherence levels of 100% and 45% to allow us to assess whether learning led to changes both for degraded and undegraded stimuli. Monkeys performed around 20 trials for each stimulus at each degradation levels during each session, and we report here the results from a total of six sessions. We identified periods of fixation during free-viewing as periods as periods of at least 100 ms duration during which eye position did not change by more than 0.3°. We then marked off a region of 0.3° × 0.3° around this position and superimposed these regions for all fixations during all relevant trials. By normalizing the volume under this function to a value of 1, we created an FPM for each stimulus. We then applied a single threshold to the FPM for all stimuli and degradation levels. The threshold η was chosen to be an order of magnitude greater than the FPM value corresponding to randomly distributed eye position, i.e., to a value of η = 10 × 1/256^2^, and these areas were converted to degrees squared of visual angle (dva^2^). The thresholded FPMs shown in Figure 7 depict the regions of the FPM that passed threshold for each of the two stimuli during an example session and thus represent the foci of eye position or regions of high fixation probability for that stimulus. Because FPMs are all normalized, a small or absent thresholded FPM region indicates that eye position was distributed on the stimulus without a clear focus. Note that for familiar stimuli, thresholded FPMs were highly consistent across sessions confirming the robustness of this measure.

#### Data analysis.

Neural activity was assessed during a fixed period of 310 ms duration, beginning 50 ms after the onset of the visual stimulus to take response latency into account. Such a period roughly corresponds to the time between saccades during natural viewing conditions. Out of a total population of 116 neurons, 83 task-related neurons were identified as showing significant differences in activity between any of the eight stimuli at any coherence level using a Bonferroni-corrected *t*-test evaluated at *p* < 0.05. Mean firing rates, reported in Table 1, were computed using the preferred stimulus for each neuron.

To assess whether learning had any systematic effect on the amount of stimulus-specific information communicated by V4 neurons, we quantified how much information was contained in the pattern of neural firing rates about novel and familiar stimuli separately. This quantity is given by the mutual information between the set of four familiar or novel stimuli and the set of associated firing rates (Shannon 1948). We thus computed the mutual information (I) among the set of stimuli (*s*) and the neural responses (*r*):



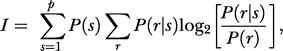



where *P*(*s*) is the probability of showing stimulus *s*, *P*(*r*|*s*) is the probability of observing a response *r* when stimulus *s* is presented, and *P*(*r*) is the probability of observing response *r*.

Because calculation of information requires many trials, we computed information for two conditions: degraded and undegraded stimuli. For degraded stimuli, we pooled the coherence levels from 35% to 65%. For undegraded stimuli, we estimated the mutual information for 100% coherence stimuli during the sample period as well as during the probe period on nonmatch trials (to exclude possible movement-related activity). We report here estimates during the probe period because they are based more trials, but results were similar for the sample period. This ensured that information measures for degraded and undegraded stimuli were based in a similar number of trials.

For each neuron we estimated four different information values, describing how much stimulus-specific information was contained in its firing rate distributions about undegraded, as well as degraded, familiar (I_fam,100_, I_fam,degrad_) and novel (I_nov,100_, I_nov,degrad_) stimuli. Note that although across all sessions we employed many more novel than familiar stimuli, each individual neuron from which we recorded during a given session ‘saw' exactly the same number of four familiar and four novel stimuli. We identified highly selective neurons in each population by selecting the 25% neurons that communicated most stimulus information about either novel or familiar stimuli (*n* = 21 out of 83 neurons total); i.e., we chose the top 25% of the distribution max(I_fam_,I_nov_). We did this because, owing to our unbiased procedure, our sample contains neurons that did not communicate large amounts of information, and we thus wanted to establish that our conclusions also applied to the neurons that communicated most information. These neurons are shown as white filled circles in Figure 3A and 3C, whereas the remaining 75% of neurons (*n* = 62) are shown as gray filled circles. There was significant overlap (13/21, 62%) between the populations of informative neurons for degraded and undegraded stimuli (χ^2^ test, *p* < 0.05), indicating that the majority of neurons that were informative for undegraded stimuli were also informative for degraded stimuli. There were no significant differences between informative neurons and the entire population in terms of mean firing rate. Unless otherwise noted, we used paired *t*-tests to compare information measures obtained for novel and familiar stimuli.

## Abbreviations

BOLD: blood-oxygen level dependent
DMS: delayed matching to sample
dva: degrees of visual angle
FPM: fixation probability map
IT: inferior temporal
MRI: magnetic resonance imaging
PF: prefrontal
PSTH: peri stimulus time histogram
V1: primary visual cortex
